# Robust Silica–Agarose Composite Aerogels with Interpenetrating Network Structure by In Situ Sol–Gel Process

**DOI:** 10.3390/gels8050303

**Published:** 2022-05-16

**Authors:** Xin Yang, Pengjie Jiang, Rui Xiao, Rui Fu, Yinghui Liu, Chao Ji, Qiqi Song, Changqing Miao, Hanqing Yu, Jie Gu, Yaxiong Wang, Huazheng Sai

**Affiliations:** 1 School of Chemistry and Chemical Engineering, Inner Mongolia University of Science & Technology, Baotou 014010, China; yangxin975@163.com (X.Y.); jpj1692787089@163.com (P.J.); xrnpdf@163.com (R.X.); liuyinghui0419@163.com (Y.L.); j1063708309@163.com (C.J.); songqiqiaa@163.com (Q.S.); qingmc@163.com (C.M.); qing194810@163.com (H.Y.); gujie199504182021@163.com (J.G.); wangyaxiong2021@126.com (Y.W.); 2 Inner Mongolia Key Laboratory of Coal Chemical Engineering & Comprehensive Utilization, Inner Mongolia University of Science & Technology, Baotou 014010, China; 3 Aerogel Functional Nanomaterials Laboratory, Inner Mongolia University of Science & Technology, Baotou 014010, China

**Keywords:** silica aerogels, agarose aerogel, nanocomposites, interpenetrating network, mechanical properties, thermal insulation

## Abstract

Aerogels are three-dimensional nanoporous materials with outstanding properties, especially great thermal insulation. Nevertheless, their extremely high brittleness restricts their practical application. Recently, although the mechanical properties of silica aerogels have been improved by regulating the precursor or introducing a polymer reinforcer, these preparation processes are usually tedious and time-consuming. The purpose of this study was to simplify the preparation process of these composite aerogels. A silicic acid solution treated with cation exchange resin was mixed with agarose (AG) to gel in situ, and then composite aerogels (CAs) with an interpenetrating network (IPN) structure were obtained by aging and supercritical CO_2_ fluid (SCF) drying. Compared to previous works, the presented CAs preparation process is briefer and more environmentally friendly. Moreover, the CAs exhibit a high specific surface area (420.5 m^2^/g), low thermal conductivity (28.9 mW m^−1^ K^−1^), excellent thermal insulation properties, and thermal stability. These results show that these CAs can be better used in thermal insulation.

## 1. Introduction

Aerogel is an ultraporous three-dimensional nanomaterial obtained by a special drying method of wet gel so that the liquid phase between the gel skeleton is replaced by the gas phase, and the gel skeleton remains intact [1]. Moreover, aerogels have a low density (~0.003–0.5 g/cm^3^), a high specific surface area (500–1200 m^2^/g), high porosity (80%~99.8%), and low thermal conductivity (~15 mW m^−1^ K^−1^), which have good application prospects in many fields such as separation, thermal insulation, energy storage, batteries, and aerospace [2]. However, traditional inorganic oxide (e.g., silica (SiO_2_), alumina (Al_2_O_3_), and zirconia (ZrO_2_)) aerogels have inherently high brittleness due to their pearl-necklace-like network, which limits their widespread applications [3,4]. Therefore, researchers constantly exploit a more appropriate approach to prepare silica aerogel materials with excellent mechanical properties, which is critical to meet the demand of their future practical applications [5,6,7].

To date, researchers around the world have mainly utilized precursor regulation [8] and external doping [9] to enhance the mechanical capacity of silica aerogel materials. Initially, the number of nanoparticles and their connection points in the aerogel skeleton were increased by promoting the amount of precursor, and then the strength of the aerogel was improved in a limited range [8]. Following this, Rao et al. [10] prepared flexible aerogels by supercritical drying after gelation of trialkoxysilane reagent RSiX_3_ (R is alkyl, aromatic or vinyl; X is an alkoxy group) [11,12,13]. The inert group R on the precursor inhibits the formation of rigid Si–O–Si bonds in the gel skeleton and decreases their number, so that the gel skeleton can adapt to certain deformations and show good flexibility. Moreover, researchers have used alkoxy silicon with an alkyl chain as a precursor to gel alone or with other alkoxysilane precursors to endow aerogels more flexibility [14]. Similarly, Gao et al. [15] and Guoqing Zu’s team [16,17,18] used similar flexible chain as precursors to prepare SiO_2_ aerogels materials, which greatly reduced the brittleness of the gel skeleton and allowed to obtain aerogel materials with a uniform microstructure and super flexibility. However, these expensive precursors increase the production cost of aerogels. To reduce the costs of preparation, researchers had synthesized silica composite aerogels using cheap precursors. Waterglass (sodium silicate) may be a viable and inexpensive alternative to alkoxide precursors. Therefore, sodium silicate produced from biomass ash, such as corn stalk ash [19], rice hull ash [20], groundnut hull ash [21], and fly ash [22], is potential candidate due to its low cost and facile production.

**Figure 1:**
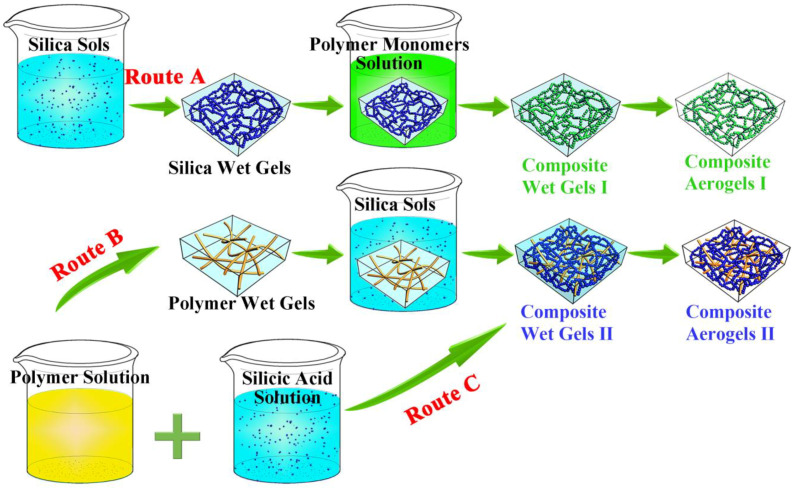
Schematic of the preparation process for composite aerogels via three different routes.

There are also some researchers who have enhanced the mechanical properties of silica aerogels by implementing various reinforcers to toughen their structure [23,24]. In early studies, the easiest way of improving the strength of silica aerogel was to use the fiber reinforcement process. Various reinforced fibrous materials in dispersed or felt materials made of fibers, such as ceramic fiber [25], polymer fiber [26], carbon fiber [27,28], glass cotton mat [29], and polyethylene terephthalate (PET) nonwoven mat [30], have been used as structural reinforcements [31]. However, these composite aerogels may have an uneven distribution of reinforcing fibers during the gelation process, or the relatively macroscopic structural reinforcements in size are incompatible with the nanoscale gel skeleton of silica. Recently, improving the mechanical properties of silica aerogels by polymer coating on the silica gel skeleton or depositing silica nanoparticles on the fibrous polymer matrix has attracted more attention [32]. On the one hand, the silica sols were prepared by the sol–gel method to prepare the silica wet gel, and immersed in the polymer monomer (e.g., methyl methacrylate [33], styrene [34], isocyanate [35], and epoxy [36]), which induced polymerization on the surface of the silica gel skeleton to form an isomorphic coating structure (composite aerogel I obtained from route A in Figure 1). The isomorphic covering method can effectively strengthen the aerogel skeleton, especially the joining of secondary nanoparticles. However, this has also brought about some shortcomings or new problems; for instance, the high cost of preparation and the formation of polymers on the surface of the gel skeleton will fill the voids between primary nanoparticles. This phenomenon shows that the specific surface area of aerogels is significantly reduced, which also limits their application in some fields. On the other hand, the polymer solution has been gelled to obtain polymer wet gels, and the polymer wet gels have been impregnated with silica sols to make the silica sol gelation form an interpenetrating network (IPN) structure (composite aerogel II route B in Figure 1) [37,38,39]. Although the mechanical properties of silica have been improved by constructing flexible polymer substrates via the above two methods, the diffusion of polymer monomers into the silica gel skeleton or the diffusion of silica sols into the polymer matrix are both time-consuming [40] and their preparation conditions are harsh (e.g., gelation at a low temperature to inhibit the gelation of sols before they diffuse into the polymer matrix [41]).

This study aimed to directly mix a polymer solution (i.e., AG solution) with a silicic acid solution in situ to construct composite aerogels II with an IPN structure, as shown in route C in Figure 1 and Figure S1. In the preparation process of CAs, AG first gelled to construct a nanofiber network, and then a SiO_2_ gel skeleton was formed in the AG nanofibers network in situ to form an IPN structure. Compared to other works [40,42], the construction of a composite aerogel gel skeleton did not require diffusion of one monomer into another, which extremely simplified the preparation process. Moreover, the CAs benefited from the fact that the gelation process of the natural polymer AG without any chemical cross-linking agents and AG nanofibers in the IPN structure of the CAs were more loosely distributed. Then, the CAs were prepared using inorganic Na_2_O·3SiO_2_ instead of organic TEOS as the silica source and compounded with AG in situ. This preparation process not only cut down the cost but also made the compound process more environmentally friendly. More importantly, the presented CAs displayed excellent mechanical properties, thermal insulation, and thermal stability, thus promoting the application of aerogels in thermal insulation.

## 2. Results and Discussion

### 2.1. Microstructures Characterization

**Figure 2:**
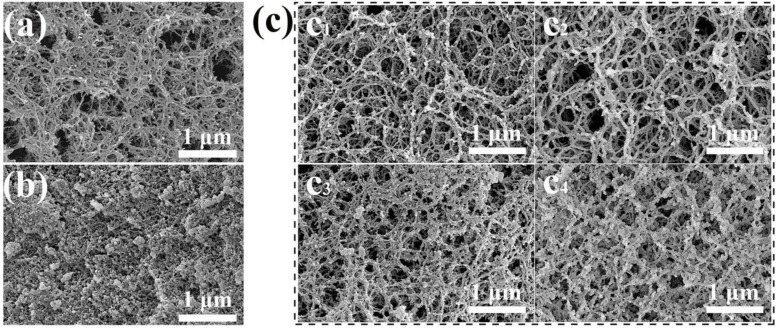
SEM images of AA-2 (**a**) SA-4, (**b**) and CAs (**c**) (CA-1 (**c_1_**), CA-2 (**c_2_**), CA-3 (**c_3_**), and CA-4 (**c_4_**), respectively).

The microstructures of agarose aerogel (AA-2), silica aerogel (SA-4), and CAs were revealed by scanning electron microscopy (SEM) images (Figure 2). The 3D network structure of AA-2 consists of disordered and dispersed nanoscale AG nanofibers (Figure 2a). The branching points were firmly fixed, and a 3D network structure was formed by hydrogen bonding or electrostatic attraction between the helical AG molecules and the flexible chains [43,44]. An SEM image of the SA-4 is given in Figure 2b. It can be seen that micrometer-sized SiO_2_ agglomerations with a typical aerogel structure were produced for pure SAs. However, during the gelation process of the CAs, AG first formed a gel skeleton due to the low-temperature self-coagulation of AG, and SiO_2_ gelled in the gel skeleton of AG to form an IPN network structure. SEM images of the CAs showed that the IPN structure was formed by a flexible AG and a rigid SiO_2_ gel skeleton (Figure 2c). CA-1 was mainly composed of AG nanofiber aggregates when the content of SiO_2_ in the composite solution was low, with a handful of SiO_2_ nanoparticles attached to the gel skeleton of AG by hydrogen bonding (Figure 2c_1_). For larger contents of SiO_2_, as in CA-2 and CA-3, a SiO_2_ gel skeleton was gradually formed in the AG nanofiber network (Figure 2c_2_,c_3_). This phenomenon indicates that a nanoscale IPN structure was constructed. When the content of SiO_2_ in the CAs was improved further, the SiO_2_ gel skeleton became denser, as shown in CA-4 (Figure 2c_4_). Compared to other composite aerogels enhanced with short fibers in a dispersed [26] or cellulose nanofiber network [45], the AG nanofibers were more evenly distributed in the CA network. Moreover, the IPN structure of the CAs made full use of these strong link points to prevent them separating from each other to maintain the macroscopic integrity of the sample.

**Figure 3:**
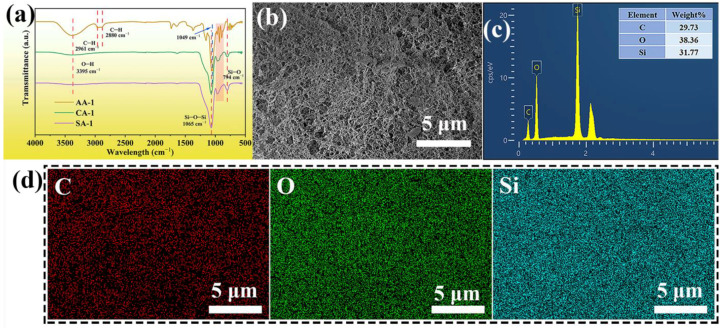
(**a**) ATR-FTIR spectra of AA-1, CA-1, and SA-1. SEM images, (**b**) weight concentration from EDS, (**c**) and EDS elemental mapping images (**d**) for the C, O, and Si elements of CA-2.

Attenuated total reflection Fourier transform infrared (ATR-FTIR) spectra of the samples are presented in Figure 3a. In the ATR-FTIR spectrum of AAs, the characteristic peaks at 3395 cm^−1^ and 1049 cm^−1^ indicate the O–H stretching vibration and glycoside stretching bond, respectively [44,46]. The peaks appeared at 2961 and 2880 cm^−1^ could be corresponded to symmetric and the asymmetric vibrations of C–H groups, respectively [47]. There were emerging peaks for CAs at around 1065, 961, and 794 cm^−1^, attributed to Si–O–Si stretching vibration, Si–OH bending vibration, and Si–O stretching vibration [48], which was not found in the ATR-FTIR spectra of the AAs. Similarly, these characteristic peaks also appeared in the SAs. This phenomenon shows that the CAs had Si–O–Si bonds that formed a gel skeleton of silica, and Si–O bonds were formed by the reaction between SiO_2_ and the AG surface. Moreover, ATR-FTIR spectrum of CA-1 and SA-1 was almost similar. This phenomenon could be because of SiO_2_ nanoparticles were tightly attached to the surface of the AG nanofibers during the formation of the SiO_2_ gel skeleton, which made the weak absorption peak of AAs difficult to observe in the ATR-FTIR spectrum of the CAs. Moreover, strong peak at 1049 cm^−1^ (glycoside stretching bond) for AA-1 and characteristic peak at 1065 cm^−1^ (Si–O–Si) for CA-1 were almost overlapping in the ATR-FTIR spectra. Thus, it is impossible to distinguish in the CAs. However, in the 930~960 cm^−1^ (in the red box of Figure 3), the CAs have the common characteristic peak of both AAs and SAs, which indicates that the CAs are composite of AG and SiO_2_. Therefore, the peak of O–H at 3395 cm^−1^ of the CAs was weaker than that of the AAs. This further indicates that silica was successfully formed in the AG skeleton and combined with AG in CAs. In conclusion, ATR-FTIR spectra analysis proved that CAs had successfully been prepared.

According to the microstructure of CA-2 (Figure 3b), energy-dispersive X-ray spectra (EDS) were captured to investigate the weight concentration and element distribution of CA-2 (Figure 3c,d). AA-1 showed carbon and oxygen peaks without silicon peaks (Figure S2), while SA-1 displayed silicon and oxygen peaks (Figure S3). However, there were not only carbon and oxygen peaks but also silicon peaks in the EDS of CA-2 (Figure 3c). These appearances further indicate that successful combination of SiO_2_ and AG. From the EDS mapping images (Figure 3d), it can be seen that the distribution of Si in CA-2 was relatively even, which also illustrates that the gel skeleton of silica was relatively uniformly distributed in the CAs.

### 2.2. Nitrogen Adsorption–Desorption Test

**Figure 4:**
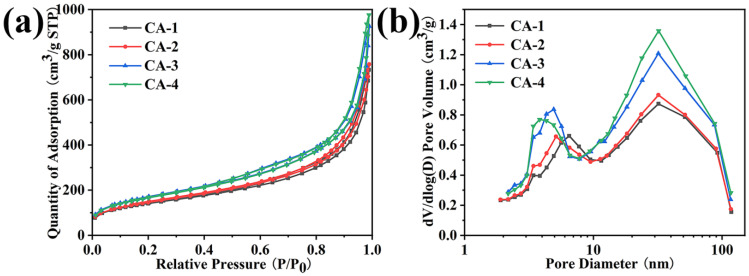
(**a**) N_2_ adsorption–desorption isotherms and (**b**) BJH pore size distributions of the CAs samples.

The N_2_ adsorption–desorption curves, Barrett–Joyner–Halenda (BJH) pore size distributions, specific surface areas, and average pore sizes of the CAs, AAs, and SAs are shown in Figure 4 and Figure S4 and Table 1. In the N_2_ adsorption–desorption isotherm, the CAs, AAs, and SAs showed a type IV isotherm of the hysteresis loop [49,50], indicating the formation of mesoporous structure (Figure 4a and Figure S4a,c). The hysteresis loops of the CAs became more obvious as the SiO_2_ concentration increased. With the decrease in the concentration of AG, the N_2_ adsorption of the AAs decreased, while the SAs increased with the increase in the SiO_2_ content. This phenomenon also proves that the N_2_ adsorption of the CAs gradually increased with the increase in the SiO_2_ content. Remarkably, the most probable distribution of the pore size of the CAs was mainly around 30 nm, and the diameter of a small amount of the pores was between 3 and 7 nm in the BJH pore size distributions (Figure 4b), which also occurred in the SAs (Figure S4d). Although the BJH pore size distributions showed that the CAs had a mesoporous structure to a certain extent, the total mesoscale pore volume of the CAs was relatively higher than that of the AAs (Table 1 and Figure S4b) and lower than that of the SAs (Figure S4d). The pore volume of the CAs was slight increase with the increase in SiO_2_ content (Table 1). These results could be due to some of the SiO_2_ nanoparticles tightly adhering to the AG nanofibers to restrain the formation of a mesoporous structure, which was in agreement with the SEM analysis. Moreover, with the increase in SiO_2_ concentration, the density of the CAs increased to a certain extent and the porosity slightly decreased owing to the formation of SiO_2_ gel skeleton. The specific surface areas of the CAs gradually increased from 272.4 to 420.5 m^2^/g with an increase in the SiO_2_ content according to the results of a BET test (Table 1). Compared to the other silica-based aerogels, the CAs showed lower density and larger specific surface area (Table 2). On the one hand, when the concentration of SiO_2_ was low, the specific surface area of the CAs was a little higher than that of the natural AAs due to many SiO_2_ aggregates without interconnection and was attached in the AG nanofibers network to increase the AG surface roughness (Table 1). On the other hand, a greater and rougher SiO_2_ gel skeleton formed in the AG nanofiber network as the concentration of SiO_2_ increased further, which made the specific surface areas of the CAs gradually increased. The average desorption pore size (10.5–11.8 nm) of the CAs is presented in Table 1, showing almost the same trend as the specific surface area, while the average desorption pore size showed a smaller range of variation than the specific surface area because of it only reflecting the pore size of the mesoporous range according to the BET analysis. Therefore, the content of SiO_2_ has an important impact on the gel skeleton of AG–SiO_2_ composite aerogels.

### 2.3. Mechanical Properties

**Figure 5:**
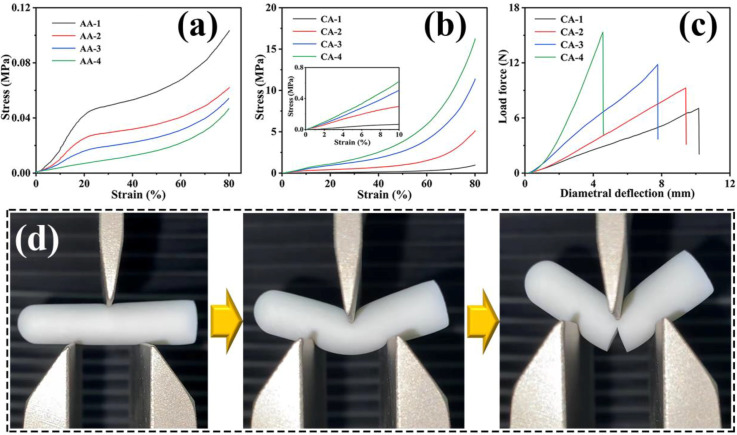
Compressive stress–strain curves of (**a**) AAs and (**b**) CAs (inset of magnification of the part within 10% strain). (**c**) Force–diametral deflection curves of the three-point bending tests on CAs. (**d**) Photographs of a three-point bending test for CA-1.

Sufficient mechanical strength plays a significant role in the application of thermal insulation materials. The mechanical properties of the prepared CAs were studied by a compressive test and a three-point bending test. Amusingly, brittle SAs combined with extremely soft AAs could produce CAs with an amazing pliability and compressive property. The mechanical properties of the AAs prepared with different concentrations of AG are presented in Figure 5a. The AAs could withstand compressive stress over 80%. When the strain was less than 20%, the compressive stress of the AAs with a low concentration increased at a slow rate, while those AAs with a higher concentration increased more rapidly. This explains that the gel skeleton of the AAs with a higher concentration gradually became more compact, which was equivalent to reducing the space of deformation. It is worth noting that the AAs did not have an obviously brittle point at higher stress. This was mainly because the gel skeleton of the AAs, with flexibility itself, can easily be deformed without breaking when it was impacted by external force. In significant contrast to the brittleness of traditional SAs, the CAs were compressed to more than 80% strains without cracks, demonstrating remarkable flexibility and nonbrittleness (Figure 5b). All of the stress–strain curves of the CAs displayed three stages, including a linear elastic district at low strain values (below 10%), a plastic district with a relatively gently growth curve at middling strain values (from 10% to 60% strain), and the final densification stage with a rapidly rose stress at high strain values (over 60% strain) [56,57]. The compression modulus of the CAs gradually increased at a low SiO_2_ content, and then dramatically increased to 6.23 MPa for CA-4 (Table 1). It is worth mentioning that the compression modulus of the CAs is higher than that of some silica-based aerogels, such as silica/polyimide (SiO_2_/PI) nanocomposite aerogel (1.9 MPa), silica nanotube aerogels (0.3~1.9 MPa), and hydrophobic silica-based aerogel (0.2 MPa) (Table 2). This further confirmed the excellent mechanical properties of the CAs prepared by compounding AG and silica. In particular, the dramatic increase in compressive stress with a high concentration was caused by the formation of more rigid gel skeletons of SiO_2_ in the AG nanofibers network to resist the impact of external forces more effectively. Therefore, by using this synthesized in situ method, CAs with the IPN structure can be obtained by relying on the SiO_2_ concentration. Furthermore, the CAs were able to withstand a large diametral deformation (i.e., approximately 4–10 mm) without breaking and the breaking force was in the range of around 6–10 N, as proved in the three-point bending tests with a fixture span of 15 mm (Figure 5c). During this process, a crack of CA-1 appeared just after 10 mm diametral deformation as shown in Figure 5d. This indicates that the CAs had good flexibility. When the concentration of SiO_2_ gradually increased, the diametral deformation gradually decreased. This may be because the free movement of the AG nanofiber was restricted when more and denser rigid gel skeletons were formed in AG nanofibers network, which resulted in the compression of the free deformation space of AG nanofiber network [41].

### 2.4. Thermal Insulation Performance

The CAs displayed low thermal conductivity, which was almost similar to native SAs [58] (Table 1). It is slightly superior to those of traditional insulating materials such as polymer foam (20~50 mW m^−1^ K^−1^) and mineral wool (35~80 mW m^−1^ K^−1^) [18] and is competitive to those of the similar silica-based aerogels [9,23,55,56,57,58] at a room temperature (Table 2). For the four kinds of CAs with different SiO_2_ content explored in this work, the thermal conductivity of those CAs increased to some extent with the increased of SiO_2_ content. This could be understood as the gel skeleton of the CAs being mainly composed of a large number of AG nanofibers with a loose microstructure, so that CA-1 showed relatively lower thermal conductivity. When the concentration of SiO_2_ further increased (CA-3 and CA-4), more and denser gel skeletons of SiO_2_ were formed in the AG nanofibers, leading to a density increase and a decrease in porosity. This could increase the heat transfer caused by contact between the solids, resulting in slightly increasing the thermal conductivity. However, the thermal conductivity of the CAs was still low because of their mesoporous structure. Therefore, the concentration of SiO_2_ is critical to prepare CAs in terms of their thermal insulation properties.

**Figure 6:**
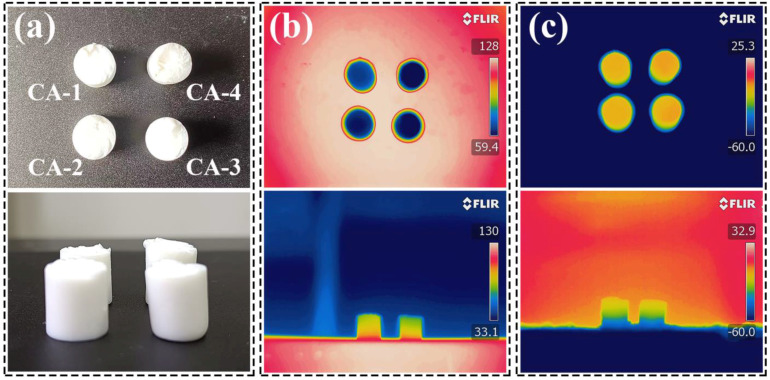
(**a**) Optical photo of the main and side views, respectively, of the CAs. FLIR images of the CAs (**b**) on the heating base plate at 130 °C and (**c**) on an aluminum plate of dry ice (–60 °C) for the main and side views, respectively.

To further prove the thermal insulating performances, the surface temperature difference of the CAs during the heating and cooling processes was recorded using an infrared thermal camera as shown in Figure 6 and Figure S5. Optical photos of the CAs of the main and side views are shown in Figure 6a and Figure S5a. The temperature difference change of the CAs at different heat source temperatures (60, 90, 110, and 130 °C) in the same view is shown in Figure 6b and Figure S5b, which show that the temperature difference gradually increased as the hot plate gradually increased, and the temperature difference could reach 68 °C when the temperature was close to 130 °C. Although the thermal conductivity of CA-1 was relatively low at room temperature, the infrared photo of CA-1 did not show a good thermal insulation effect compared to the other CAs at a high temperature. This may be because the high porosity of CA-1, but the lack of mesoporous content that can more effectively inhibit the diffusion of air molecules, resulting in poor thermal insulation after heating. With the increase in the solid concentration, the gradual formation of more SiO_2_ gel frameworks in the AG nanofiber network could inhibit the heat transfer more effectively. In the meantime, the temperature difference of the CAs was approximately 40–50 °C at a low temperature (–60 °C) as shown in Figure 6c and Figure S5c. This illustrates that the CAs had excellent thermal insulation properties, whether in a high- or low-temperature environment.

### 2.5. Thermal Stability

**Figure 7:**
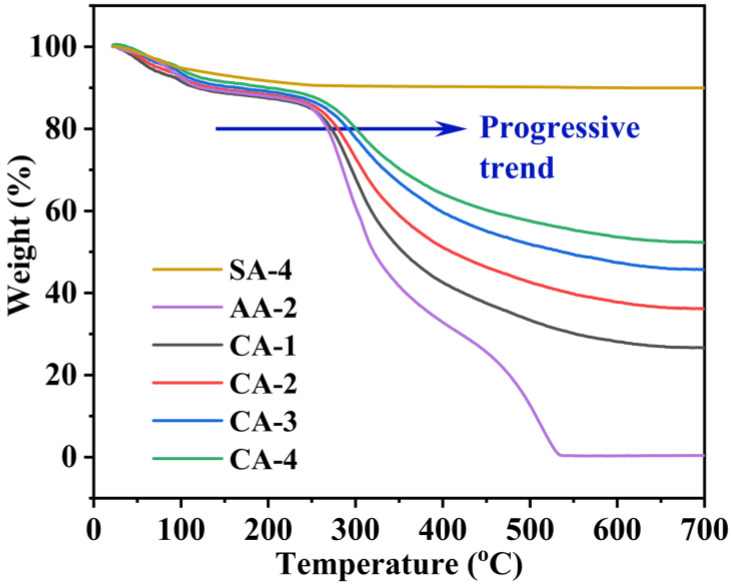
The TGA curve of SA-4, AA-2, and the CAs.

Outstanding thermal stability of materials is crucial for their thermal insulation application, so the thermal stability of the materials was determined by thermogravimetric analysis (TGA), as shown in Figure 7. First, SA-4, AA-2, and the CAs lost approximately 10% of their weight at 30–250 °C. This may be because of the adsorption of water molecules on the sample gel skeleton surface [7,58]. However, the weight loss of SA-4 at 250–700 °C was eventually maintained at 10%. Moreover, the temperature of decomposition of AG in the CAs gradually shifted from approximately 250 °C to approximately 260 °C (Figure 7) as the SiO_2_ concentration increased. When the weight gradually decreased to 80% of the initial weight, as shown by the dark blue arrow in Figure 7, the temperature corresponding to the CAs gradually increased from 260 °C to approximately 300 °C as the SiO_2_ content increased. Therefore, SiO_2_ was able to restrain decomposition of the AG gel skeleton, which improved the thermal stability of the CAs. Furthermore, AA-2 completely degraded at approximately 510 °C and lost almost 100% of its weight. Nevertheless, the CAs completely degraded at approximately 650 °C and lost approximately 25%–50% of their weight. This illustrates that more and denser SiO_2_ gel skeletons formed in the AG nanofibers network as the concentration of SiO_2_ increased, which is beneficial for improving the thermal stability of CAs.

## 3. Conclusions

In this work, robust composite aerogels were prepared by an AG solution mixed with a silicate solution in situ to gradually form an IPN structure. In the gelation process of the CAs, the flexible AG firstly gelled to construct a nanofiber network due to AG’s self-gelling feature, and then a rigid SiO_2_ gel skeleton was formed in the loose AG nanofibers network to form an IPN structure. Compared to other polymer toughening technologies that can effectively improve aerogels, the time-consuming diffusion of a reaction substance into other wet gels was not involved here, which made the preparation process simpler and more efficient. Furthermore, cheaper water glass (Na_2_O·3SiO_2_) was used to instead of traditional TEOS as a silicon source, which greatly reduced the costs and was environmentally friendly. In addition, the CAs displayed a low density (0.079 g/cm^3^), high porosity (96.0%), high specific surface area (as high as 420.5 m^2^/g), and excellent mechanical properties. Especially, the CAs also exhibited good thermal insulation properties and thermal stability, which is important for its application in the field of thermal insulation. This work offers a novel and facile design of composite aerogels, which also provides new ideas for the preparation of high-performance composite aerogels.

## 4. Materials and Methods

### 4.1. Materials

First, 732 cation exchange resin and sodium silicate (Na_2_O·3SiO_2_) were purchased from Macklin Biochemical Co., Ltd. (Shanghai, China). Agarose (AG) was bought from Beijing Wobisen Technology Co., Ltd. (Beijing, China). Sulfuric acid (H_2_SO_4_), sodium hydroxide (NaOH), ammonium hydroxide (NH_3_·H_2_O) and ethanol were obtained from Beijing Chemical Reagent Co., Ltd. (Beijing, China). All chemicals were of analytical grade and were used as received without further purification.

### 4.2. Preprocessing of 732 Cation Exchange Resin

First, the strong acid styrene 732 cation exchange resins was washed with hot water at 70–80 °C to remove impurities and make the effluent colorless. Next, the cation exchange resin in the exchange column was soaked in 1 mol/L of H_2_SO_4_ (2 times the amount of resin) for 2 h, rinsed with 1 mol/L of H_2_SO_4_ (4 times the amount of resin), and washed to neutral with deionized water.

### 4.3. Preparation of AG–SiO_2_ Composite Aerogels

First, the sodium silicate solutions were poured into the excess cation exchange resin to remove Na^+^ in the exchange column to obtain silicic acid solutions (pH = 2~3). The silicic acid solution (SiO_2_ in solution, 10% *w*/*w*) and AG solution (3% *w*/*w*) were stirred to mix at 80 °C with different volume ratios of AG to silicic acid solution (1:1, 1:2, 1:3, and 1:4), and their pH was adjusted to 5~6 by 0.1 mol/L of NH_3_·H_2_O (less than 0.2 μL). Then, the mixed sols were poured into Petri dishes (90 mm) or centrifuge tubes (5 mL) to form AG–SiO_2_ composite gels. Those composite gels in the Petri dishes with a diameter of 90 mm were cut into 15 mm × 15 mm × 20 mm cubes by blade. The composite gels were aged in a mixture of ethanol and water at 50 °C for 1.5 h to harden the silica gel skeleton and completely replace the liquid in the composite wet gels with ethanol. The composite wet gels were dried by supercritical CO_2_ fluid at a flow rate of 5–15 L/h at 11 MPa and 40 °C for 2.5 h to obtain AG–SiO_2_ composite aerogels, labeled as CA-1, CA-2, CA-3, and CA-4 according to volume ratio of AG and silicate mixture solution (1:1, 1:2, 1:3, or 1:4).

### 4.4. Preparation of AAs

The preparation process of the AAs was similar to that of the CAs, with only the silicic acid solutions being replaced by deionized water. In the first place, different concentrations of the AG solutions were prepared by mixing the AG solution (3% *w*/*w*) with deionized water in a microwave at different volume ratios (1:1, 1:2, 1:3, and 1:4). Then, the AG solutions were poured into a Petri dish with a diameter of 90 mm and cooled naturally at room temperature to prepare AG gel. Next, the AG hydrogels were cut into 15 mm × 15 mm × 20 mm cubes by a blade. Finally, the AG hydrogels were replaced by anhydrous ethanol solvents and dried by SCF to obtain AAs, labeled as AA-1, AA-2, AA-3, and AA-4 according to the concentrate of AG (1.5%, 1.0%, 0.75%, or 0.6%), as shown in Figure S6.

### 4.5. Preparation of SAs

The preparation process of the SAs was similar to that of the CAs, with only the AG solutions being replaced by deionized water. First, different concentrations of silicic the acid solution was prepared by mixing silicic acid solution (10% *w*/*w*) with deionized water at different volume ratios (1:1, 1:2, 1:3, and 1:4). Then, the silicic acid solutions were adjusted to pH = 5~6 by 0.1 mol/L of NH_3_·H_2_O (less than 0.2 μL) and poured into centrifuge tubes to achieve gelation. Next, the SiO_2_ gels were aged in a mixture of ethanol and water at 50 °C for 1.5 h to harden the silica gel skeleton. Finally, SAs were obtained by the ethanol solution replacement method and SCF drying, labeled as SA-1, SA-2, SA-3, and SA-4 according to the concentrate of SiO_2_ (5%, 6.7%, 7.5%, or 8%), as shown in Figure S7.

### 4.6. Characterization

The micromorphology, functional groups (FTIR), pore size distributions, density, porosity, and the specific surface areas of the CAs were determined. The mechanical properties, thermal insulation properties and thermal stability were also evaluated. The detailed characterization methods are provided in the Supplementary Materials. The abbreviations in the work are shown in Table 3.

## Figures and Tables

**Table 1:** Physical properties of composite aerogels (CAs), agarose aerogels (AAs), and silica aerogels (SAs).

Sample	Bulk Density (g/cm^3^)	Porosity ^a^ (%)	S_BET_ (m^2^/g)	Average Pore Size ^b^ (nm)	Pore Volume ^c^ (cm^3^/g)	Compression Modulus (MPa)	Thermal Conductivity (mW m^−1^ K^−1^)
CA-1	0.079	96.0	272.4	10.5	0.78	0.68	28.9
CA-2	0.107	94.8	304.8	10.4	0.86	2.90	30.5
CA-3	0.123	94.0	375.3	11.1	1.21	5.05	32.3
CA-4	0.128	93.8	420.5	11.8	1.32	6.23	34.6
AA-1	0.029	98.4	269.1	13.6	0.88	0.21	32.2
AA-2	0.021	98.8	239.0	14.1	0.76	0.13	30.4
AA-3	0.019	98.9	227.8	14.2	0.65	0.08	28.7
AA-4	0.018	99.0	219.9	14.3	0.60	0.04	26.3
SA-1	0.062	97.0	742.3	13.8	2.55	d	e
SA-2	0.089	95.8	754.8	14.8	2.61	d	e
SA-3	0.107	94.9	767.0	14.9	3.06	d	e
SA-4	0.116	94.5	839.0	16.7	3.25	d	e

^a^ The porosity includes both mesopores and all void space. ^b^ Mean pore diameter determined using a nitrogen desorption branch and BJH. ^c^ Pore volume is the single point pore volume with p/p_0_ = 0.985 during BET test. d, e: The related parameters could not be measured or calculated because SAs are extremely fragile.

**Table 2:** Comparison of the properties of the CAs and other silica-based aerogel materials.

Materials	Density (g/cm^3^)	S_BET_ (m^2^/g)	Pore Volume (cm^3^/g)	Compression Modulus (MPa)	Thermal Conductivity (mW m^−1^ K^−1^)	Ref.
AG–SiO_2_ composite aerogel	0.079~0.128	272.4~420.5	0.73~1.09	0.68~6.23	28.9~34.6	This work
polyurethane foam	not reported	not reported	not reported	not reported	20~50	[18]
mineral wool	not reported	not reported	not reported	not reported	35~80	[18]
SiO_2_/PI nanocomposite aerogel	not reported	not reported	not reported	1.9	31.1~41.6	[51]
SiO_2_–SSNF aerogel	0.085~0.093	not reported	not reported	30~70	25~29	[9]
silica nanotube aerogels	0.025	327~427	0.99~1.15	0.3~1.9	30.2~32.6	[23]
fumed silica insulation	0.5~1.2	not reported	not reported	0.15	33	[52]
ZrO_2_ fiber/GF and fumed SiO_2_/Al_2_O_3_	0.733~0.761	not reported	0.04~0.05	0.02~0.18	50~77	[53]
hydrophobic silica-based aerogel	0.047~0.077	28.4~337.0	0.059~0.267	0.2	24	[54]
silica aerogels blanket/ board	0.08~0.2	600~800	not reported	not reported	≥15	[55]

**Table 3:** Abbreviations and full names of the work.

Abbreviations	Full Names	Abbreviations	Full Names
AG	agarose	BJH	Barrett–Joyner–Halenda
SiO_2_	silica	TGA	thermogravimetric analysis
CAs	composite aerogels	SEM	scanning electron microscopy
AAs	agarose aerogels	ATR-FTIR	attenuated total reflection fourier transform infrared
SAs	silica aerogels	EDS	energy-dispersive X-ray spectra
SSNF	SiO_2_/SnO_2_ nanofibers	SCF	supercritical CO_2_ fluid
GF	glass fiber	TEOS	tetraethyl orthosilicate
PI	polyimide	IPN	Interpenetrating network
BET	Brunner−Emmet−Teller	PET	polyethylene terephthalate

## Data Availability

The data presented in this study are available on request from the corresponding author.

## Supplementary Materials

The following are available online at https://www.mdpi.com/article/10.3390/gels8050303/s1: Figure S1. Flowchart illustrating the overall processes used in this work; Figure S2. SEM images (a), weight concentration from EDS (b) and EDS elemental mapping images (c) for C and O elements of the AA-1; Figure S3. SEM images (a), weight concentration from EDS (b) and EDS elemental mapping images (c) for O and Si elements of the SA-1; Figure S4. (a) N_2_ adsorption-desorption isotherms and (b) BJH pore-size distribution of AAs. (c) N_2_ adsorption-desorption isotherms and (d) BJH pore-size distribution of SAs; Figure S5. (a) Optical photo at main and side views, respectively, of the CAs. FLIR images of the CAs (b) on the heating base plate at different temperatures (60, 90, 110, and 130 °C) and (c) on aluminum plate of dry ice (–60 °C) at main and side views, respectively.; Figure S6. Image of the AAs; Figure S7. Image of the SAs.
