# Supplementary material for: Robust Silica–Agarose Composite Aerogels with Interpenetrating Network Structure by In Situ Sol–Gel Process
Source: Gels. 2022 May 16;8(5):303. doi: 10.3390/gels8050303 (PMC9141877; doi:10.3390/gels8050303)
Supplement: Supplementary file 1 [file gels-08-00303-s001.zip › gels-1678590-supplementary.pdf]

gels

Article

Robust Silica–Agarose Composite Aerogels with Interpenetrating Network Structure by In Situ Sol–Gel Process

Xin Yang [1,2,3], Pengjie Jiang [1,2,3], Rui Xiao [1,2], Rui Fu [1,2,3,*], Yinghui Liu [1,2,3], Chao Ji [1,2], Qiqi Song [1,2,3], Changqing Miao [1,2,3], Hanqing Yu [1,2,3], Jie Gu [1,2,3], Yaxiong Wang [1,2,3] and Huazheng Sai [1,2,3,*]

[1] School of Chemistry and Chemical Engineering, Inner Mongolia University of Science & Technology, Baotou 014010, China; yangxin975@163.com (X.Y.); jpj1692787089@163.com (P.J.); xrnpdf@163.com (R.X.); liuyinghui0419@163.com (Y.L.); j1063708309@163.com (C.J.); songqiqiaa@163.com (Q.S.); qingmc@163.com (C.M.); qing194810@163.com (H.Y.); gujie199504182021@163.com (J.G.); wangyaxiong2021@126.com (Y.W.)

[2] Inner Mongolia Key Laboratory of Coal Chemical Engineering & Comprehensive Utilization, Inner Mongolia University of Science & Technology, Baotou 014010, China

[3] Aerogel Functional Nanomaterials Laboratory, Inner Mongolia University of Science & Technology, Baotou 014010, China

* Correspondence: furui14@mails.ucas.edu.cn (R.F.); shz15@tsinghua.org.cn (H.S.)

Citation: Yang, X.; Jiang, P.; Xiao, R.; Fu, R.; Liu, Y.; Ji, C.; Song, Q.; Miao, C.; Yu, H.; Gu, J.; et al. Robust Silica–Agarose Composite Aerogels with Interpenetrating Network Structure by In Situ Sol–Gel Process. *Gels* **2022**, *8*, 303. https://doi.org/10.3390/gels8050303

Academic Editors: Jannis Wernery and Samuel Brunner

Received: 28 March 2022
Accepted: 10 May 2022
Published: 16 May 2022

Publisher's Note: MDPI stays neutral with regard to jurisdictional claims in published maps and institutional affiliations.

Copyright: © 2022 by the authors. Submitted for possible open access publication under the terms and conditions of the Creative Commons Attribution (CC BY) license (https://creativecommons.org/licenses/by/4.0/).

Supplementary Materials

1. Characterization

1.1. Morphology and Nanostructure

Field-emission scanning electron microscope (SEM) characterization of the CAs, AAs and SAs was performed by a Thermo Scientific Apreo 2C (Waltham, Massachusetts, American) at accelerating voltage of 10 kV and a working distance of 10 mm. These samples were analyzed by simply sticking on the sample holder using a carbon pad, followed by coating platinum.

1.2. Energy-Dispersive X-ray Spectra (EDS)

The relative elements content of the CAs, AAs and SAs were determined by EDS. The test conditions were nearly the same as those used for SEM characterization, yet the samples were thicker.

1.3. Attenuated Total Reflection Fourier Transform Infrared (ATR-FTIR) Spectroscopy Analysis

ATR-FTIR infrared spectroscopy analysis was performed to evaluate the chemical changes the samples. The samples with about 5 mm× 5 mm× 5 mm cubes were pressed into slice. The ATR-FTIR spectra of the samples were obtained by a PerkinElmer (Spectrum 3, Waltham, Massachusetts, American). The scan range of ATR-FTIR spectroscopy was 500–4000 cm^{-1}.

1.4. Density

The density of CAs, AAs and SAs was determined by measuring the weight and volume of each individual sample. The weight of each aerogel block was measured using an analytical balance (readability 0.0001 g, Sartorius). The dimensions of each aerogel block were measured by a digital caliper. Five blocks were used for density determination for each sample and averaged.

1.5. Content of silica in CAs

The content of silica in CAs was calculated as according to Equation S1, where m_s and m_a are the mass of silica and AG nanofibers in the CAs, respectively; $\rho_{CA\text{-}n}$ and $\rho_{AA\text{-}n}$ are the bulk density of CAs and AAs corresponding to each other.

$$\text{Mass fraction (\%)} = \frac{m_s}{m} = \frac{m - m_a}{m} = \frac{\rho_{CA\text{-n}} - \rho_{\text{AA-n}}}{\rho_{\text{CA-n}}} \tag{S1}$$

1.6. Porosity

The porosity (P) of the CAs was calculated according to Equation S2, where ρ, ρ_s and ρ_a are the bulk density of CAs, the skeleton densities of pure SAs and AAs; ω_s and ω_a were the mass fraction of silica and AG in CAs, respectively. Herein, the ρ_s and ρ_a were fixed at 2.1 g/cm^3 and 1.8 g/cm^3.

$$\text{P (\%)} = \left(1 - \frac{\rho}{\omega_s\rho_s + \omega_a\rho_a}\right) \times 100\% \tag{S2}$$

The porosity (P) of the AAs was calculated according to Equation S3, where ρ and ρ_a are the bulk density of AAs, the skeleton densities of pure AAs, respectively. Herein, the ρ_a was fixed at 1.8 g/cm^3.

$$\text{P (\%)} = \left(1 - \frac{\rho}{\rho_a}\right) \times 100\% \tag{S3}$$

The porosity (P) of the SAs was calculated according to Equation S4, where ρ and ρ_s are the bulk density of SAs, the skeleton densities of pure SAs, respectively. Herein, the ρ_s was fixed at 2.1 g/cm^3.

$$\text{P (\%)} = \left(1 - \frac{\rho}{\rho_s}\right) \times 100\% \tag{S4}$$

1.7. Nitrogen Physisorption Measurements

The samples (~30 mg) were measured the nitrogen adsorption and desorption isotherm, pore size distribution, and specific surface area at 77 K by physical adsorption instrument (ASAP 2460, Shanghai, China). The samples were first outgassed at 130 °C for 5 h before measuring. The amount of N_2 absorbed at various relative vapor pressures (nine points where the pressure is $0.05 < p/p_0 < 0.3$, the cross-sectional area of nitrogen molecules is calculated as 0.162 nm^2) was used to determine the surface area by Brunauer-Emmett-Teller (BET) analysis method. The pore size distribution of the relevant samples was obtained in the adsorption-desorption curves by the Barrett-Joyner-Halenda (BJH) algorithm.

1.8. Thermal conductivity measurements

Thermal conductivity of the AAs and CAs was measured by a C-Therm TCi thermal conductivity analyzer (Fredericton, New Brunswick, Canada) through a transient plate method. All the samples were cut to 5 mm thickness and put on the transient plane source, and then a weight was put on the sample to make sure the contact between the sample and the transient plane was complete.

1.9. Thermal Stability

The thermal degradation behavior of the CAs and AAs in nitrogen was analyzed and investigated by thermogravimetry analyzer (TGA, STA449 F3). A sample weight of approximately 2 mg was used and heated from 30 to 700 °C at a heating rate of 10 K/min in a ceramic pan.

1.10. Thermal Insulation Measurement

The infrared imaging was gained by a thermal infrared camera (FLIR T620, USA). The camera was operated at a distance of about 10–30 cm. The samples were put on the heating plate or aluminum plate of dry ice. The samples were tested after 10 min of remaining at a constant surface temperature.

1.11. Mechanical Properties

The compression tests were conducted on HD-B609B-S (Guangdong, China). The CAs and AAs with the approximately 15 × 15 × 20 mm cubes were employed, while the compressive rate was set as 6 mm/min. Moreover, The CAs blocks with approximately 15 × 15 × 20 mm were employed during the three-point bending crack test.

2. Flowchart of CAs

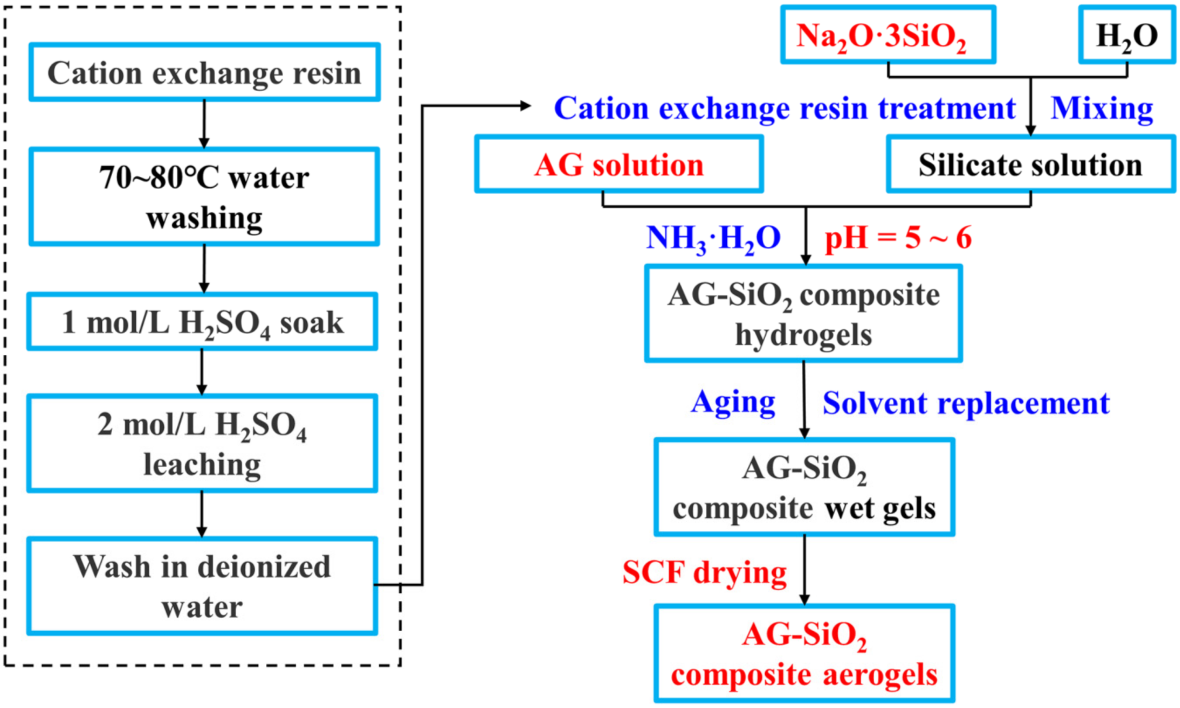

Figure S1. Flowchart illustrating the overall processes used in this work.

3. EDS of AA-1

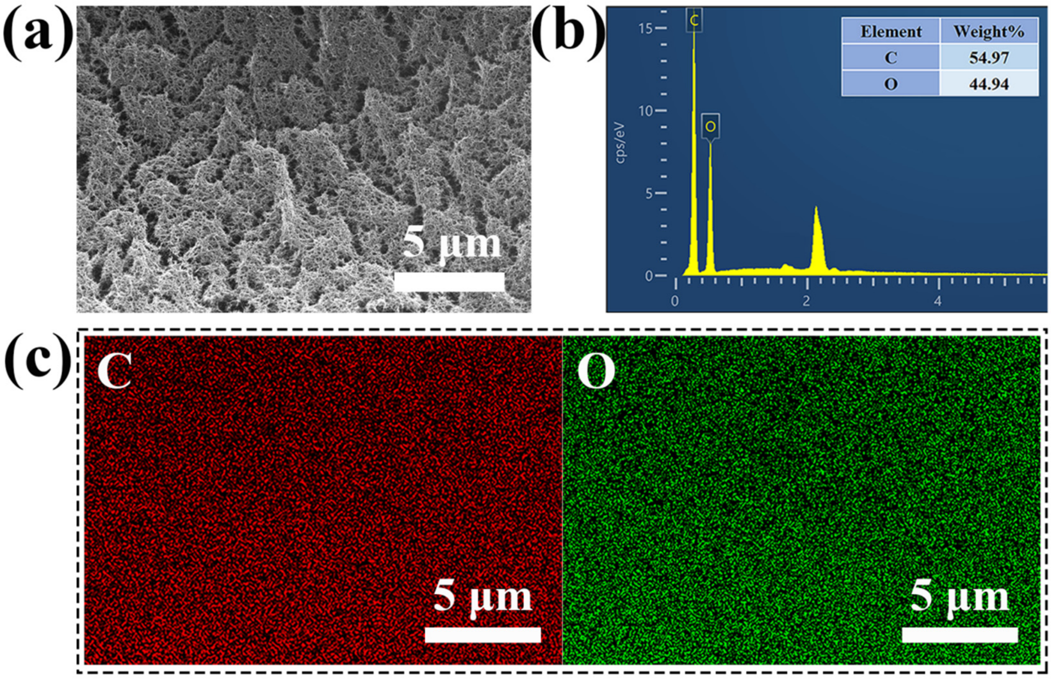

Figure S2. (**a**) SEM images, (**b**) weight concentration from EDS and (**c**) EDS elemental mapping images for C and O elements of the AA-1.

4. EDS of SA-1

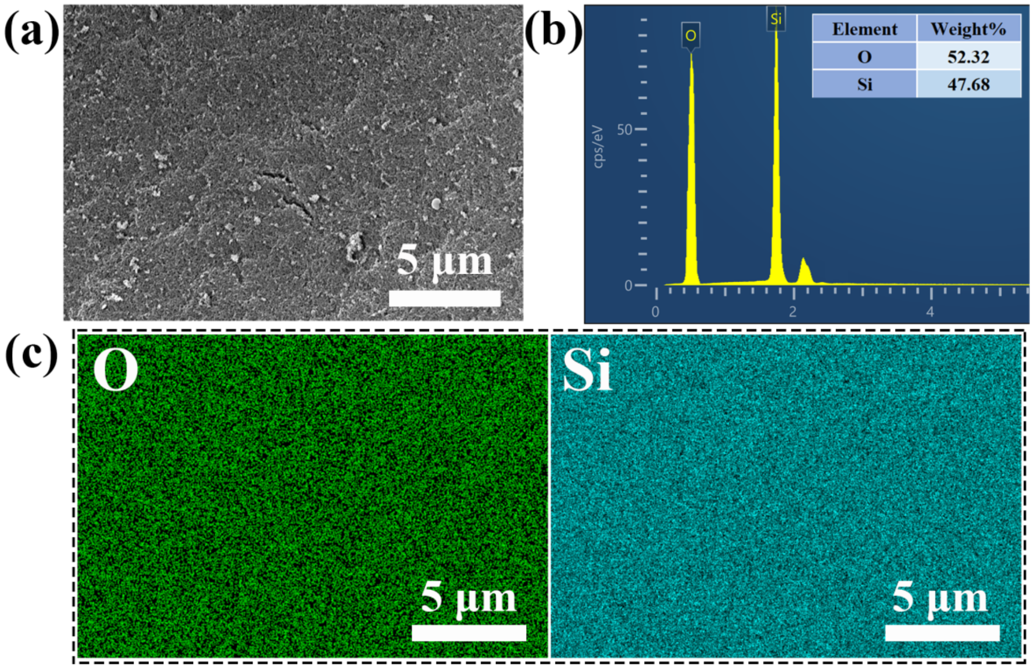

Figure S3. (**a**) SEM images, (**b**) weight concentration from EDS and (**c**) EDS elemental mapping images for O and Si elements of the SA-1.

5. Nitrogen Adsorption–Desorption Test of AAs and SAs

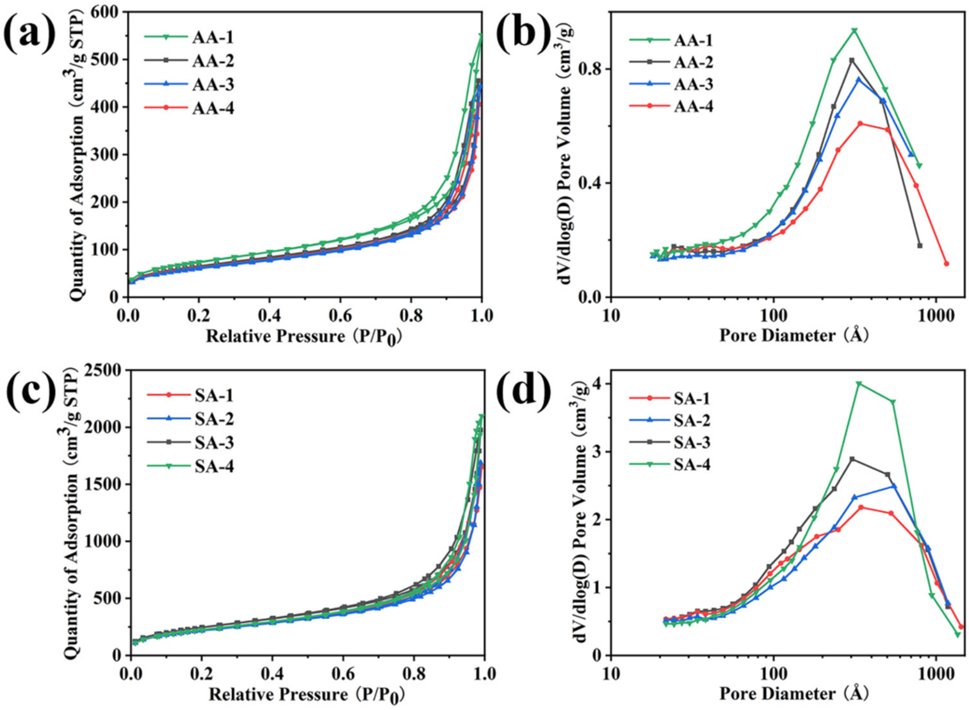

Figure S4. (**a**) N_2 adsorption-desorption isotherms and (**b**) BJH pore-size distribution of AAs. (**c**) N_2 adsorption-desorption isotherms and (**d**) BJH pore-size distribution of SAs.

6. Optical Photos and Infrared Photos of CAs

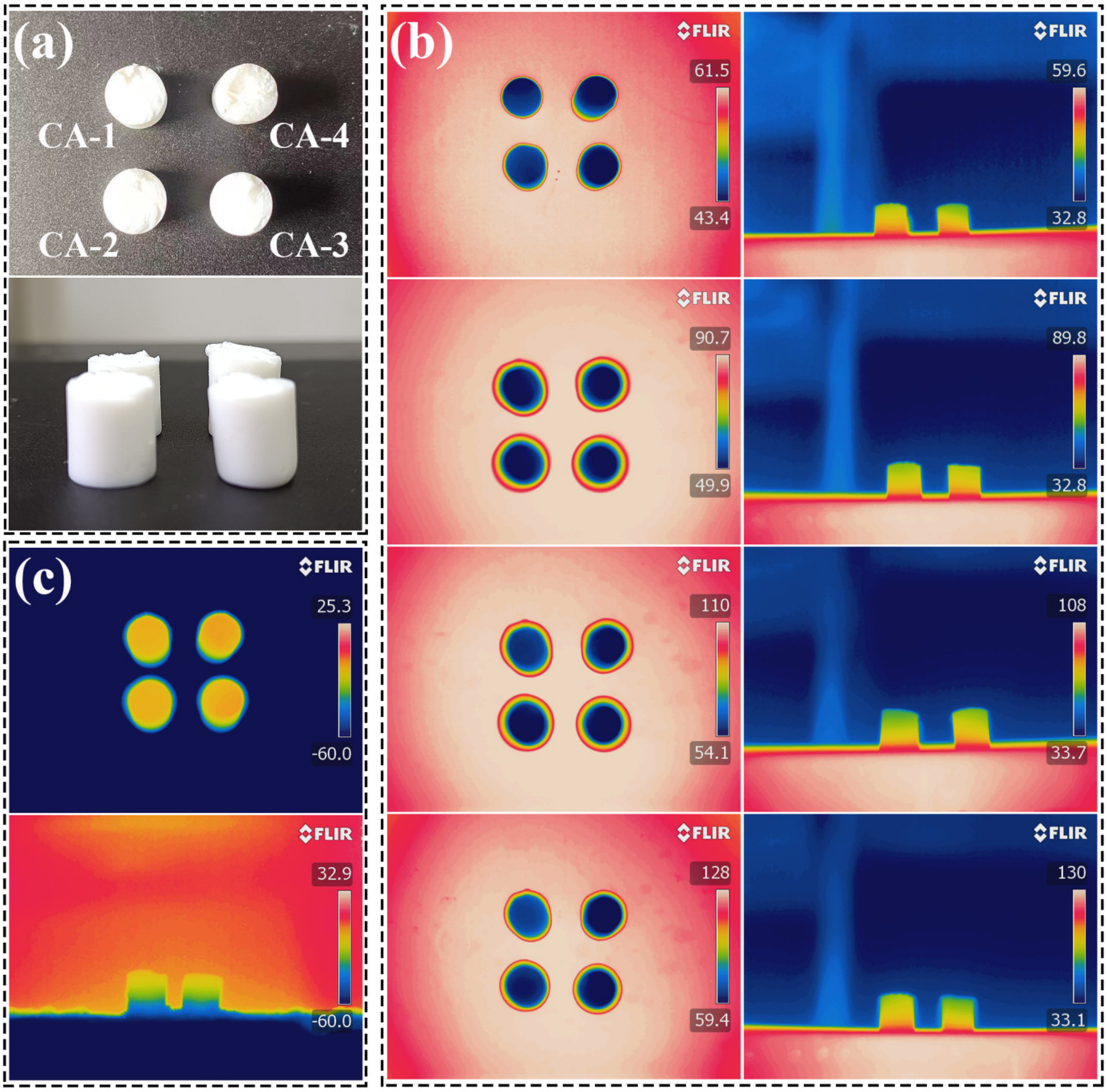

Figure S5. (**a**) Optical photo at main and side views, respectively, of the CAs. FLIR images of the CAs (**b**) on the heating base plate at different temperatures (60, 90, 110, and 130 °C) and (**c**) on aluminum plate of dry ice (−60 °C) at main and side views, respectively.

7. Image of AAs

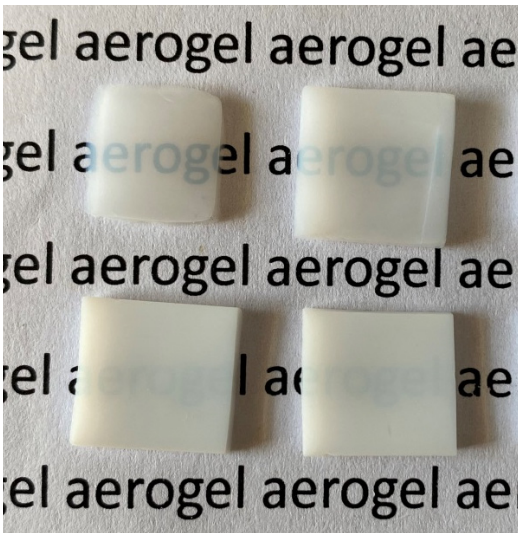

Figure S6. Image of the AAs.

8. Image of SAs

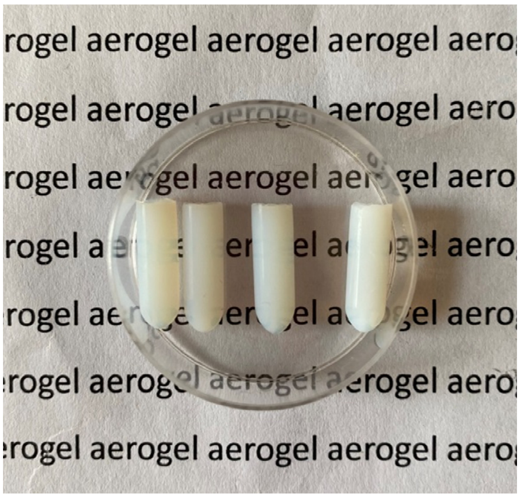

Figure S7. Image of the SAs.
